# Chronic pain, chronic stress and substance use: overlapping mechanisms and implications

**DOI:** 10.3389/fpain.2023.1145934

**Published:** 2023-06-21

**Authors:** J. Schaffer, N. Fogelman, D. Seo, R. Sinha

**Affiliations:** Department of Psychiatry and the Yale Stress Center, Yale University School of Medicine, New Haven, CT, United States

**Keywords:** stress, chronic pain, ventromedial prefrontal cortex, sex differences, substance use

## Abstract

Chronic pain is among the most common reasons adults in the U.S. seek medical care. Despite chronic pain's substantial impact on individuals' physical, emotional, and financial wellness, the biologic underpinnings of chronic pain remain incompletely understood. Such deleterious impact on an individuals' wellness is also manifested in the substantial co-occurrence of chronic stress with chronic pain. However, whether chronic stress and adversity and related alcohol and substance misuse increases risk of developing chronic pain, and, if so, what the overlapping psychobiological processes are, is not well understood. Individuals suffering with chronic pain find alleviation through prescription opioids as well as non-prescribed cannabis, alcohol, and other drugs to control pain, and use of these substances have grown significantly. Substance misuse also increases experience of chronic stress. Thus, given the evidence showing a strong correlation between chronic stress and chronic pain, we aim to review and identify overlapping factors and processes. We first explore the predisposing factors and psychologic features common to both conditions. This is followed by examining the overlapping neural circuitry of pain and stress in order to trace a common pathophysiologic processes for the development of chronic pain and its link to substance use. Based on the previous literature and our own findings, we propose a critical role for ventromedial prefrontal cortex dysfunction, an overlapping brain area associated with the regulation of both pain and stress that is also affected by substance use, as key in the risk of developing chronic pain. Finally, we identify the need for future research in exploring the role of medial prefrontal circuits in chronic pain pathology. Critically, in order to alleviate the enormous burden of chronic pain without exacerbating the co-occurring substance misuse crisis, we emphasize the need to find better approaches to treat and prevent chronic pain.

## 1. Introduction

While 1 in 5 Americans suffer with chronic pain (1), the understanding of why and how chronic pain develops remains ambiguous. Chronic pain is defined as pain that persists or recurs for over 3 months [per International Classification of Diseases, ICD-10 (2)] or 6 months [per National Pain Strategy and NIH Task Force (1, 3)]. Chronic pain significantly impacts individuals' ability to work and maintain relationships and is significantly associated with emotional distress or depressive symptoms (4–7). In the clinical setting, opioid analgesics remain the mainstay of chronic pain treatment (8), despite evidence that chronic opioid treatment for chronic pain is not an effective pain management approach and development of novel therapies for pain and chronic pain is a national priority (9). In patients whose chronic pain motivates them to use prescription opioids for pain relief, there is an increased prevalence of comorbid mental health conditions including substance misuse and substance use disorders, emotional difficulties (e.g., depression, anxiety) and even suicidal ideation (10, 11). The prevalence and toll of chronic pain implore us to better understand the patterns and underlying pathophysiology of chronic pain in order to alleviate its burden.

## 2. Key features of chronic pain

We first look at patterns in chronic pain from an epidemiological perspective. Across studies, it has been shown that women are significantly more likely to develop chronic pain than men for certain types of pain syndromes (12–15). Additionally, mood and anxiety disorders commonly co-occur with chronic pain (4), and substance misuse tends to heighten individuals' experiences of pain. Exploring these features of the chronic pain population may help in identifying potential mechanisms that underly the development of chronic pain and uncover important targets for intervention.

### 2.1. Sex differences

There is a greater prevalence of chronic pain in women vs. in men in certain types of pain conditions (12). This greater prevalence of chronic pain in women has been reported for the following conditions: back pain, migraine, musculoskeletal pain, neuropathic pain, oral pain, osteoarthritis, and widespread pain (16). Such evidence suggests that evaluating sex differences in chronic pain development and experience can help parse potential biologic and psychologic underpinnings of chronic pain.

Animal models have shown the existence of physiological differences between males and females that affect pain sensation (14). For example, relative to male animals, females have slower recovery following chronic constriction injury, have earlier pain presentation in a model of femoral cancer, and have more fatigue-induced hypersensitivity to pain (17–19). Furthermore, female mice require more morphine to achieve an equal analgesic effect to male mice (20). This sex difference in opioid analgesic tolerance in preclinical studies is consistent with findings in clinical studies of women and men (21).

Previous studies have shown sex differences in the endogenous opioid system and the cannabinoid system (22, 23). Both pathways are involved in processing pain, pain coping and self-regulation of pain (22, 24). It has been shown that female sex is associated with lesser activation of anti-nociceptive signaling through mu opioid receptors, which could mediate their greater sensitivity to pain (25). Studies on sex differences in the endocannabinoid system reveal significantly greater CB1 activation in female vs. male hippocampus (26), greater sensitivity in response to cannabinoid ligand in females than males (27), and faster development of tolerance to cannabinoid ligands in females vs. males (28). Changes in protein expression in the endogenous cannabinoid pathway coincide with development of chronic pain conditions (29). In addition, the endocannabinoid system is also involved in stress regulation and coping (30) and animal studies have shown that increased levels of the two main endocannabinoids—2-Arachidonoylglycerol (2-AG) and anandamide (AEA)—promote resilient coping following stress (31, 32).

### 2.2. Role of chronic stress in vulnerability for chronic pain

Chronic stress has been shown to predict development of chronic pain (33, 34). Chronic stress occurs when an individual experiences sustained emotional or physiological challenges continuously over a significant period of time, leading to “wear and tear on the body” (35). Long-term stress has been shown to sensitize individuals to pain, a phenomenon known as stress-induced hyperalgesia (36–39). Bolstering this hypothesis, Ide et al. showed that unpredictable chronic mild stress reduced the pain-relieving effects of morphine in mice (40). Clinically, it has been shown (41) that a greater number of adverse events increases the risk for chronic pain development, as well as the experience of chronic stress. Furthermore, recent evidence suggests a biological link between stress experience and pain; individuals with a history of Adverse Childhood Experience (ACE) were found to have specific epigenetic changes involving a gene associated with setting individual pain thresholds (42). However, the relationship between chronic stress, chronic pain, and relationship of both to opioid use has not been explored within the same samples and we present secondary data below to illustrate this association.

### 2.3. Chronic stress and pain co-occurrence in a large community sample

In a large sample of 947 young-mid age community adults [18–55 years of age; 56% women; mean age 30.7 (s.d. = 9.95) years] who were not acutely ill psychiatrically or medically, and did not have current or past opioid use disorder (OUD) as assessed by the Structured Clinical Interview for DSM-IVTR [SCID-I (43)], we assessed chronic stress using the Cumulative Adversity Index, a structured interview assessment of cumulative adversity and stressful life events [CAI (44)], and also number of pain symptoms using the Cornell Medical Index (45). The Chronic Stress Subscale of the Cumulative Adversity Interview (CAI) consisted of 62 items relating to the subjective experience of continuous stressors or ongoing stressful life events and problems. Items were rated as not true, some to very true for perceived difficulties with specific ongoing interpersonal, social, and financial relationships and responsibilities including difficulties in the work and home environment and relationships with family and significant others. The Cumulative Adversity Interview and its chronic stress subscale has high reliability ranging from an overall 0.86 and 0.82 for the chronic stress subscale (46).

Cornell Medical Index is a questionnaire that poses “yes” or “no” questions about individuals' current occurrence of physical and emotional health symptoms, including specific types of pain symptoms. In addition to current symptoms, it also asks subjects to indicate whether they have been diagnosed with specific illnesses, and about health habits, like smoking (45). The CMI is verified across multiple studies as a good indicator of general health (47, 48).

**Figure 1:**
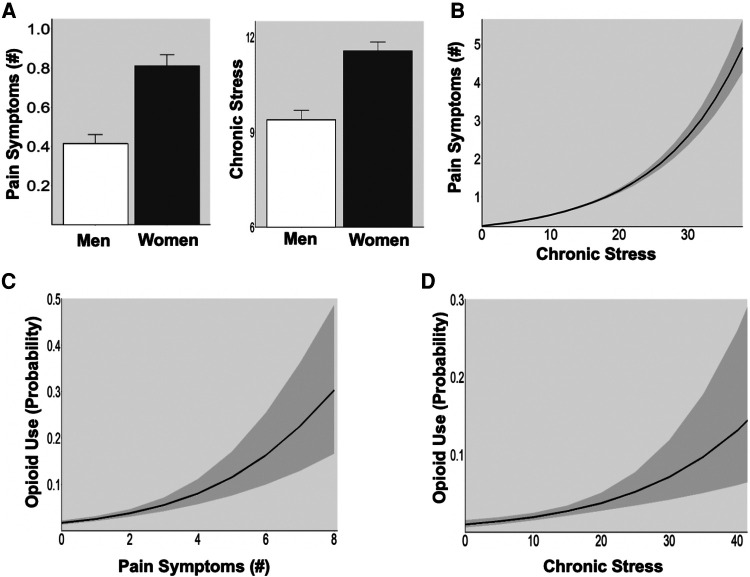
(**A**) Sex difference in pain symptoms and chronic stress. Women showed greater pain symptoms (*p* < 0.001) and higher chronic stress (*p* < 0.001) than men in a large community sample (*N* = 947). (**B**) Association between stress and pain. High chronic stress was associated with greater pain symptoms in all participants (*p* < 0.001). (**C**) Pain and opioid use. The probability of opioid use increased with greater pain symptoms (*p* < 0.001). (**D**) Stress and opioid use. The probability of opioid use increased with higher levels of chronic stress (*p* < 0.001).

In this large community sample, we found that women reported greater average number of pain symptoms (*t* = 5.6, *p* < 0.001) and higher levels of chronic stress (*t* = 5.5, *p* < 0.001) compared to men (Figure 1A). In all participants, higher chronic stress was positively associated with greater pain symptoms [Figure 1B; incidence rate ratio (IRR) = 1.08, *p* < 0.001]. Further, greater likelihood of taking opioids was predicted by both greater number of pain symptoms (Opioids: OR = 1.52, *p* < 0.026) and high chronic stress (Opioids: OR = 1.58, *p* < 0.001) (Figure 1C).

These patterns of high co-occurrence of chronic stress with chronic pain are consistent with previously cited research on the association between stress and chronic pain and further highlight that each is associated with an increased risk of opioid use in a large community sample. Moreover, female sex was associated with experiencing more chronic stress and pain, supporting previously reported vulnerability of women to related chronic stress and pain dysfunction and association with opioid misuse. While these are cross-sectional data from a large non-clinical community sample, they point to a need to further examine the basis of the associations between chronic pain and chronic stress in a longitudinal manner and assess sex differences in order to further understand the mechanisms underlying the development of chronic pain.

### 2.4. Comorbidities with negative affect and depressive disorders

It has been well-established that chronic stress can produce long-term emotional distress (49–51). As there is a positive correlation between chronic stress and physical distress, it is important to explore the interaction between incidence of long term emotional and physical pain. Data from the World Mental Health surveys show that pain conditions strongly correlate with negative affect in communities across the world (4, 5). Furthermore, Gerrits et al. have shown that individuals with current or history of anxiety or depression report experiencing more severe pain and pain in more locations than healthy controls (52). The relationship is reciprocal: within pain populations, there is greater prevalence of depressive symptoms, and within clinically depressed populations, there is more chronic pain (53). Comorbid mood disorder and chronic pain lead to poorer prognosis in both conditions than in patients with one of the two conditions alone (54). Long-term use of opioids for pain conditions correlates with an increased probability of new-onset depression (55). In the reverse direction, chronic pain patients with comorbid mood disorders are more likely to escalate long-term opioid use than chronic pain patients without mood disorders (56). This comorbidity further highlights the need to understand the emotional aspects of chronic pain in order to effectively prevent and treat chronic pain and associated mental illnesses.

### 2.5. Coping with pain and stress: relationship to pain catastrophizing

In the face of stress and pain, the methods by which different individuals cope can alleviate or aggravate pain. Over time, researchers have studied the relationship between the pain experience and catastrophizing, the latter being defined as an “exaggerated negative ‘mental set’ brought to bear during painful experiences” (57). Across pain conditions, catastrophizing proves to exacerbate intensity and emotional distress accompanying pain (57–59), and also increases the risk of developing chronic pain (60–62). Importantly, multiple sub-populations of individuals who tend to catastrophize overlap with those overrepresented in the chronic pain population. Women tend to catastrophize more than men (63, 64). Patients with depression and anxiety show higher levels of catastrophizing in the face of distress (65), and catastrophizing has been shown to play a mediating role in the relationship between depression and pain (66). Patients reporting higher adverse childhood events exhibited greater levels of catastrophizing and perceived lower confidence in their ability to cope with their pain (33). The latter highlights the need to consider domains of coping and self-regulation in exploring the neural mechanisms of chronic pain and to develop not only a greater understanding of chronic pain, but also to identify specific components that must be addressed in its treatment.

### 2.6. Chronic pain and substance misuse

Substances of abuse have been shown to induce hyperalgesia, a state of hypersensitivity to pain. In opioid misuse, specifically, the phenomenon of opioid-induced hyperalgesia has been shown across preclinical and clinical models (67). In addition to hypersensitivity to pain, periods of withdrawal from chronic substance misuse creates a state of “hyperkatifeia”, or increase in intensity of negative emotional state (68, 69). The combination of heightened pain state and worsened negative emotional state may then motivate drug use dose escalation, a path by which many patients progress from opioid use to dependence (70). Furthermore, chronic pain is associated with social isolation, and opioid use has been shown to temporarily alleviate such loneliness (11, 71). However, chronic opioid use can exacerbate social isolation, contributing to the negative emotional state that then drives opioid dependence and contribute to the drug overdose mortaility rate (71).

In addition to opioid use, chronic alcohol use sensitizes users' nociception (72). As with opioid-induced hyperalgesia, this alcohol-produced painful state has been shown to motivate escalation of alcohol use in models assessing vulnerability for alcohol use disorders (73). Similarly, when individuals with cannabis use disorder attempt to stop using cannabis, many experience withdrawal involving heightened states of emotional and physical pain that can drive cannabis dependence (74). This cyclic pattern of chronic pain leading to drug use, and drug use leading to heightened physical and emotional pain states highlights the need to develop better assessment of substance-related hyperalgesia in individuals with chronic pain. Furthermore, understanding how substance misuse may facilitate development of heightened pain states can provide insight into pathways that may mediate the development of chronic pain and how best to treat such co-morbidity.

In summary, the key features associated with development of chronic pain include chronic stress and cumulative adversity experiences, mood and anxiety comorbidity, substance misuse risk and sex differences in pain experience for certain types of pain conditions and in the development of chronic pain. Additionally, specific types of pain coping may also increase risk of development of chronic pain. Notably, adverse stressful experiences in early life or during vulnerable periods of illness or adversity may increase chronic pain via epigenetic mechanisms by changing stress genes and changes in genes that support microglia during neuronal development (75, 76). Such epigenetic changes may alter the structure and function of neural circuits involved in regulating stress and pain, thereby reducing stress coping and increasing nociception. Thus, the next section explores the overlap in functional neural mechanisms of chronic stress and chronic pain and discusses the clinical implications.

## 3. Overlapping neural circuits in chronic pain and chronic stress

Recent evidence focusing on understanding the pain experience in humans, has identified a Neurologic Pain Signature (NPS), a defined pattern of brain functional magnetic response imaging (fMRI) activity underlying the pain experience (77). Evidence shows the NPS can be divided into a nociceptive component and a self-regulatory component (78–81). While regions such as the somatosensory cortices, dorsal anterior cingulate cortex (dACC), and thalamus are known to react to noxious stimuli and to signal pain experience, fronto-striatal circuits have been shown to mediate top-down self-regulation of pain (78, 79). Specifically, the nucleus accumbens (NAc)-ventromedial prefrontal cortex (VmPFC) pathway has been shown to act as an anti-nociceptive region (80), with increased blood-oxygen-level-dependent (BOLD) activity in the VmPFC inversely relating to pain (81). Recent research has also shown significant abnormalities in functional connectivity of the VmPFC and NAc and other fronto-striatal regions during resting state in individuals with chronic pain on prescription opioids compared to drug free controls, further supporting the importance of this circuit in regulating nociception (82). Importantly, studies from animal models and clinical research show that dysfunction of the medial prefrontal cortex, a key component of this self-regulatory pathway, mediates the development and persistence of chronic pain (83–86).

**Figure 2:**
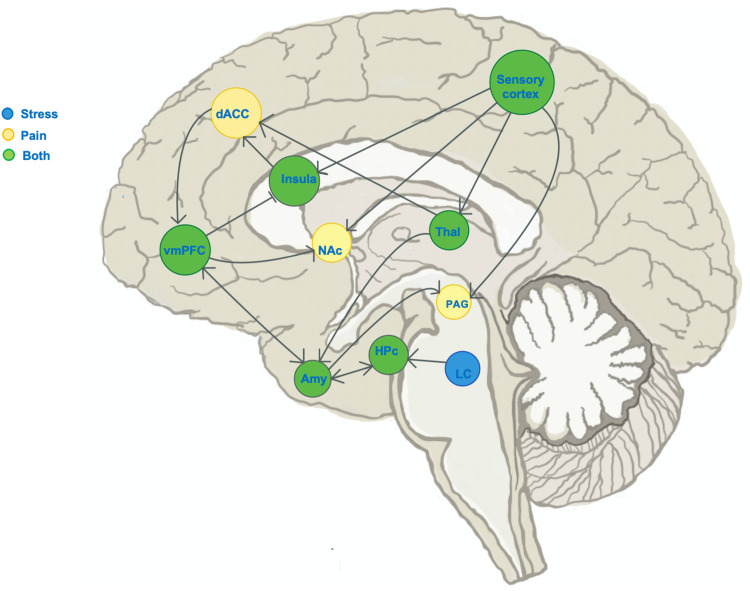
Schematic of known distinct and overlapping stress circuits. Illustration of the sagittal brain section, marking regions known to show change in activity during experience of: stress (blue), pain (yellow) or both stress and pain (green). Blunt arrowheads indicate inhibition of, while sharp arrowheads indicate stimulation of the target region by the region of arrow origin and sharp arrowheads indicate stimulation. The ventromedial prefrontal cortex (VmPFC) receives input from pain- and stress-encoding regions, and has outputs in both pain and stress pathways. dACC, dorsal anterior cingulate cortex; NAc, nucleus accumbens; Hpc, hippocampus; Thal, thalamus; LC, locus coeruleus; PAG, periaqueductal gray.

Similar to pain encoding in the brain, stress processing involves both stress-sensing components as well as top-down regulation of stress circuits and the stress experience. Acute stress activates the hypothalamic-pituitary-adrenal axis, which receives top-down regulatory input from the VmPFC (35, 87). Flexible and adaptive VmPFC function is necessary to “turn off” stress once fearful stimuli are gone (88, 89), and impaired VmPFC function is observed in patients with post-traumatic stress disorder (90). The VmPFC's role in top-down control of both emotional and physical distress makes this region a key area of interest when studying the development of chronic pain, given the simultaneous epidemiological overlaps in emotional and physical pain. We focus specifically on the self-regulatory component of pain and stress circuits because of the clear anatomical and functional overlap in brain circuits across both stress and pain. (See Figure 2 illustration of the known neural circuits of pain and stress and their overlap.)

### 3.1. Ventromedial PFC (VmPFC) and the self-regulation of pain and stress

Activity in the ventromedial prefrontal cortex has been shown to function as an anti-nociceptive neural signal (80). Chronic back pain patients were found to have reduced medial PFC (mPFC) gray matter volume (91). In a study that tasked participants with utilizing strategies to regulate their pain experience, activity in the VmPFC and nucleus accumbens were greatest when participants were actively trying to down-regulate pain (78). In a separate study of brain activity and the effects of opioid analgesia during experimental pain, baseline striatal activity was correlated with greater opioid-induced pain relief (92). As cited earlier, McConnell et al. (82) also show dysfunction in this corticostriatal circuit in chronic pain patients using prescription opioids and such dysfunction related to greater negative affect. Other work has bolstered this finding showing that using cognitive strategies that rely on mPFC circuits to distract attention from painful experiences relies on opioid-ergic networks that gate nociceptive input at the level of the spine (93). In the context of the previously noted sex differences in the endogenous opioid system, and its relation to pain processing, it will be important to explore these neurobiological circuits as it relates to predisposition to chronic pain in men and women.

If the VmPFC is necessary in self-regulation of pain, we may expect that factors known to increase the risk of developing chronic pain—like chronic stress—do so by contributing to VmPFC dysfunction. In addition to pain regulation, the VmPFC is a crucial region for adaptive coping (94) and emotion regulation (95, 96). It is involved in the self-control and regulation of emotions of both stressful (97) and rewarding nature (98). Studies on the effects of chronic stress—a known predisposing factor of chronic pain—on brain structure and function reveal that chronic stress is associated with anatomical and functional changes in the VmPFC. Chronic stress is associated with lower gray matter volume in the mPFC, VmPFC, striatum and insula (46). Chronic stress is known to increase inflammation through the peripheral and central nervous systems (76), and such inflammation is thought to contribute to the development of psychiatric disorders in patients who endured chronic stress (99). Furthermore, stress-induced inflammation is associated with disruptions in functional connectivity in the VmPFC in patients with depression (100). VmPFC and other prefrontal disruption has also been documented in patients with alcohol use disorder and substance use disorders (101–104). Taken together, these findings suggest that pathology of the VmPFC may mediate the effects of chronic stress on self-regulatory pain mechanisms.

### 3.2. Stress, pain, and hypoactive VmPFC response

In a previous study, we examined the association between cumulative adversity, including chronic stress, health symptoms and neural responses to stress vs. no-stress neutral cues in 75 healthy community adults with no history of depression, anxiety or substance use disorder (105). Cumulative adversity and chronic stress were assessed using the Cumulative Adversity Index (CAI) along with the Chronic Stress Subscale, and physical and emotional health symptoms were assessed using the Cornell Medical Index (CMI) and each of these are described in Section B3 above. In those with a history of cumulative stress and adversity, findings revealed a key neurofunctional link such that higher CAI scores corresponded to greater limbic-striatal (e.g., regions of the amygdala, hippocampus, insula, and striatum) responses to acute stress stimuli, but reduced stress-related activity in the orbitofrontal cortex (OFC), a region of the VmPFC, involved in emotion, pain, and reward self-regulation. Furthermore, hyperactivation of the hippocampus and hypoactivation of the OFC/VmPFC region was each significantly associated with greater overall number of health symptoms.

**Figure 3:**
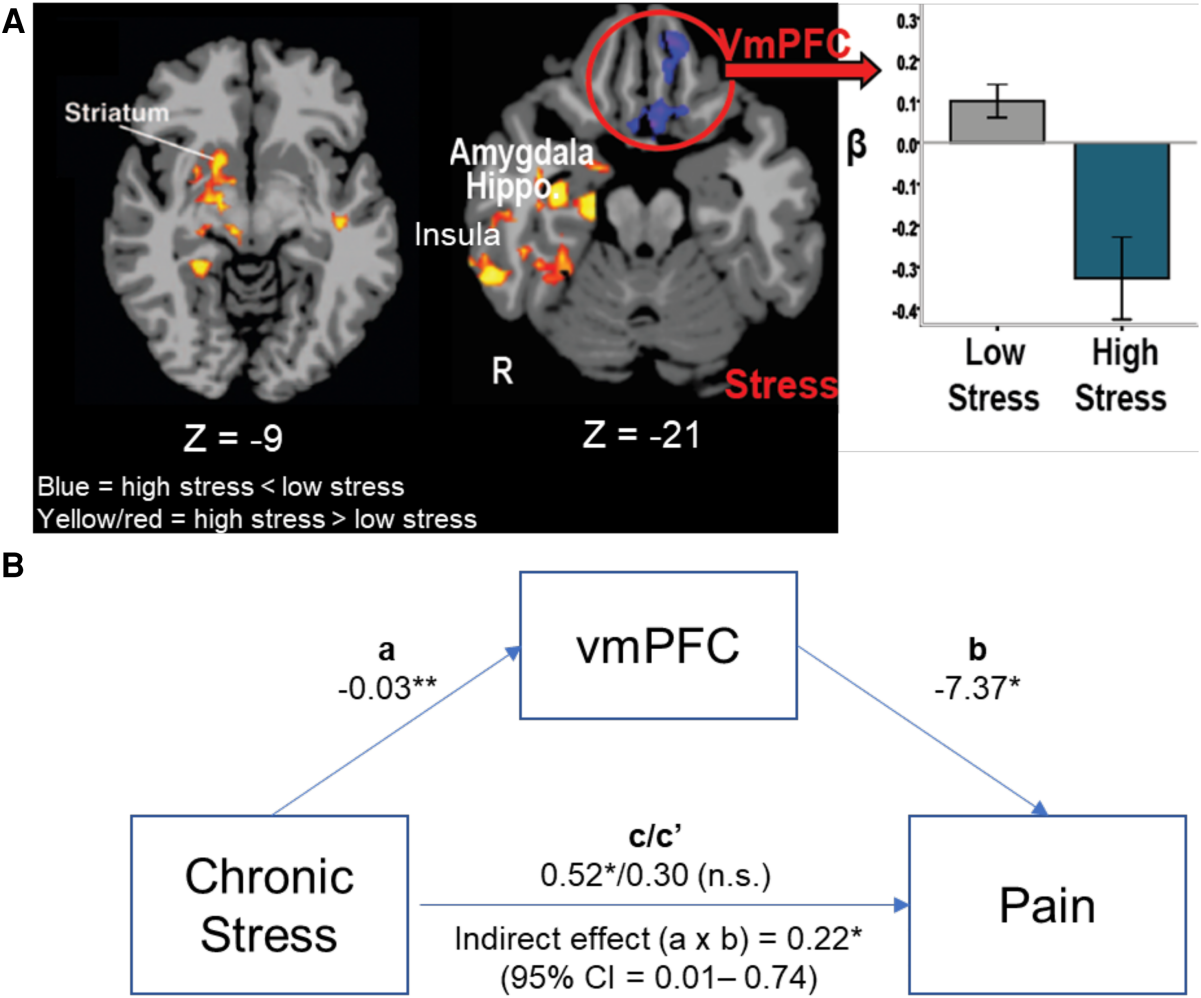
VmPFC, stress, and pain. (**A**) High Stress group (*N* = 25) show greater limbic-striatal (amygdala, hippocampus, insula and striatum) responses during acute stress exposure compared to the Low Stress (*N* = 25), but lower, more hypoactive VmPFC response to stress in the high vs. low stress groups (105). (**B**) The ROI of the hypoactive VmPFC response was entered in a mediation analysis to assess whether VmPFC functional response during acute stress mediated the effects of chronic stress on greater self reported pain symptoms (*N* = 50). Mediational analysis indicated that high chronic stress was associated with lower stress-induced VmPFC response (*a* process: −0.03, *p* < .01) and lower the VmPFC response, higher the reported pain symptoms (*b* process: −7.37, *p* < .05), and that the direct significant effect of high chronic stress associated with high pain symptoms (c = 0.52, *p* < .05) was fully accounted by the stress-induced VmPFC functional response (indirect effect *c*′: *a* × *b* = .22, *p* < .05, 95% CI = .01–.74), such that the direct effect of chronic stress on pain symptoms was no longer significant once the stress-related VmPFC response was included in the mediational mode (Direct effect *c*/*c*′ = 0.52/0.30, *p* = ns). *p < .05, **p < .01.

These findings suggest that higher levels of adversity and chronic stress may sensitize individuals to higher neural stress reactivity in emotional- and distress-sensing regions while simultaneously compromising responses of the VmPFC self-regulation region during acute stress with signiticant impact on health. This pattern is most clearly revealed in a direct comparison of those with the highest cumulative stress levels (High Stress—top one third of the sample, *N* = 25) as compared to those who report lowest cumulative stress levels (Low Stress—bottom one third of the sample, *N* = 25) as shown in Figure 3A [from Seo et al. (105)]. Furthermore, in a separate study of 30 community adults, we assessed the functioning of the VmPFC over several continuous minutes of sustained stress exposure and demonstrated that dynamic and flexible activity in the VmPFC during stress mediates active coping in stressful situations (106). Thus, it could be expected that deficient VmPFC engagement, as shown in Figure 3A, among high stress individuals may interfere with not only active stress coping, but also with active top-down regulation of pain.

To follow up on the hypothesis presented above, we conducted secondary whole brain analyses of the data presented in Seo et al. (97) to specifically examine whether the VmPFC is involved in predicting pain symptoms in the high and low stress stress groups described above, and tested its specific role in mediating the link between chronic stress and pain symptoms by conducting a mediational analysis. The VmPFC region of interest (ROI) beta values were extracted from the whole brain analysis (results shown in Figure 3A) for the High Stress and Low Stress groups (*N* = 50). The chronic stress scores from the CAI and the number of physical pain symptoms in the sample of High and Low stress groups from the Cornell Medical Index (CMI, described above in Section 2.2) were included in a mediational analysis to assess whether chronic stress predicts physical pain symptoms and whether the acute stress response of the VmPFC mediates the link between high chronic stress and higher pain symptoms. Indeed, the findings of this secondary follow-up analyses reveal that the blunted acute stress-induced VmPFC response significantly mediated the relationship between chronic stress and self-reported pain symptoms (Figure 3B).

**Figure 4:**
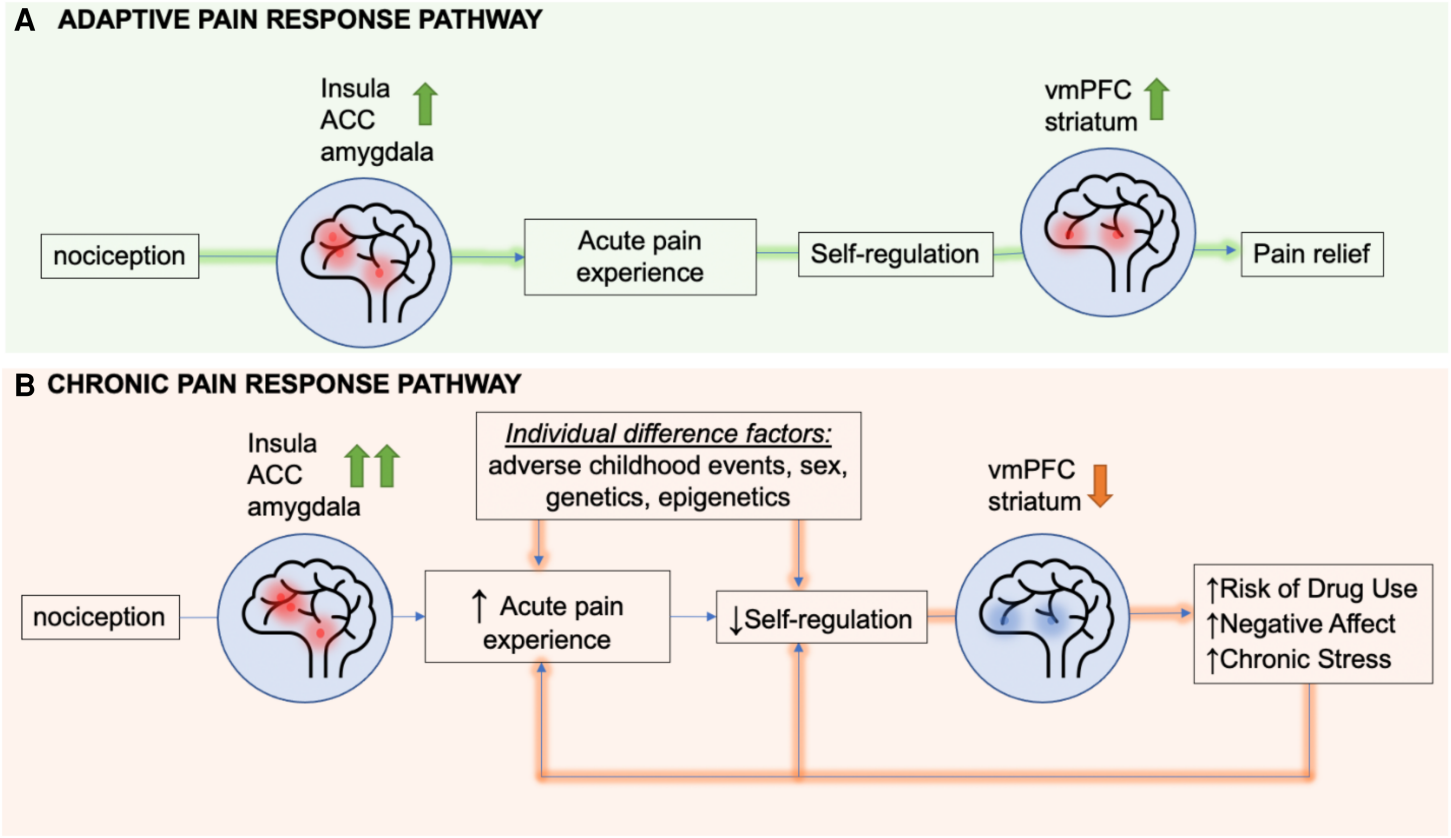
A heuristic *feed-forward* model of overlapping stress circuits and circuits driving risk of chronic pain. Presentation of a noxious stimulus results in activation of the insula, amygdala, and dorsal anterior cingulate cortex (dACC). In the adaptive pain pathway, the ventromedial prefrontal cortex (VmPFC) and striatum engage soon after the start of the acute pain experience to regulate nociceptive signals, thereby mediating pain relief. In chronic pain, factors such as adverse childhood events, sex, genes, drug use, negative affect, and stress can all increase the activity of the insula, dACC and amgydala following noxious stimulus presentation and also result in hypoactivation of the VmPFC and striatum, identified as the resilient coping and self regulation circuit. This hypoactivation impairs self-regulation of not only stress but also pain states, thereby extending the pain experience. Both heightened pain sensitivity and longer lasting pain predispose patients to risk of drug use, negative affect, and chronic stress, that in turn creates a *feed-forward sensitized* pathway towards more hypoactivation of the coping and self regulation circuit, thereby increasing risk of sustained pain symptoms and chronic pain.

These findings support the hypothesis that cummulative and chronic stress may negatively impact the VmPFC, compromising self-regulatory control over the stress-pain circuit, leading to risk of greater physical pain symptoms. Together, these findings also support the hypothesis that VmPFC may serve as a a common overlapping neural region that may underlie the high association between chronic stress and increased vulnerability to chronic pain. On the basis of this neurobiological overlap, a heuristic *feed-forward* model on the overlap of chronic pain, chronic stress and substance misuse risk is proposed in Figure 4, wherein chronic stress states alter VmPFC related circuits that regulate stress, reward and pain. A dysfunctional VmPFC circuit then results in risk of greater acute pain experience and poor self-regulation of pain that, in turn, increases the risk of sustained pain symptoms and development of chronic pain (Figure 4).

### 3.3. Summary on overlapping processes of chronic stress and chronic pain

In this section, we address the neurobiological link between pain and stress, substance use, and sex differences. Critically, the VmPFC is a neurobiological focus in the neural circuits underlying each of these conditions and appears to be a key region mediating the relationships amongst these variables. Many studies have pointed to the overlap between stress and pain circuits (7). In individuals with a history of early life stress, as well as those with current diagnoses of post-traumatic stress disorder, imaging studies show a blunted VmPFC stress response (107, 108, 109). Early life stress has been linked specifically to VmPFC hypoactivation (38, 110). Patients with alcohol use disorder show a similar VmPFC dysfunction in response to stress (101). These same circuits are also dysfunctional in patients with chronic pain using prescription opioids (82). A decrease in prefrontal activity has also been shown to be correlated with increased levels of pain catastrophizing (111). Conversely, an increase in VmPFC responding during stress has been associated with higher levels of active coping (106). Furthermore, the VmPFC is a key region regulating limbic and striatal regions, regions responsive to stress and reward stimuli respectively. As reviewed in above sections, substantial neurobiological evidence indicate that the VmPFC-limbic-striatal circuit may underlie the pathology of co-occurring chronic pain and chronic stress. Given that the VmPFC exerts regulatory control over subcortical regions involved in stress (e.g., amygdala) and reward (striatum) processing, VmPFC dysfunction in individuals with chronic pain may increase risk of common comorbid conditions including depression, anxiety, prescription opioid misuse and other substance use disorder (SUD). Initially, individuals with chronic pain may experience pain symptoms alone. However, as their symptoms worsen, they are likely to experience pain, distress, and emotional difficulties (e.g., depressed and anxious mood) resulting from sensitized VmPFC-limbic-striatal circuit governing both pain and stress as shown in Figure 4. Continued chronic pain and distress are likely to further compromise this circuit, weakening the VmPFC control over the striatal regions (reward system). The subsequently disinhibited striatal system may result in difficulties controlling an urge to use substances (e.g., opioid, alcohol, and other substances), and to cope with pain and emotional distress, further increasing the risk of other comorbid conditions including depression, anxiety, and SUD in individuals with chronic pain.

Although this pattern can occur in both men and women, given some evidence of sex differences in prevalence of chronic pain subtypes (12) and high prevalence of affective disorders in women (112, 113), it is likely that there are sex differences in the manifestation of this pathology, and therefore further research in both men and women is needed to understand pain-related dysfunction in the VmPFC-limbic-striatal circuit. In support of this, our group has shown that sex differences in stress responding are mediated by the VmPFC. While dorsomedial activity tends to attenuate the stress response in men, VmPFC activity seems to dampen stress reactivity in women (114). Thus, hypoactivity of the VmPFC in women may lead to difficulties with controlling striatal and limbic response to stress and reward stimuli, which may explain high prevalence of chronic pain, and co-occurring emotional disorders in epidemological studies.

## 4. Clinical implications

Our current pharmacological approaches to treating chronic pain target the opioid, gamma-aminobutyric acid (GABA), and cannabinoid systems (115–117). As noted earlier, opioid agonists are commonly prescribed for chronic pain conditions, but chronic opioid use itself may exacerbate chronic pain. We have reviewed findings on opioid-induced hyperalgesia, and the processes by which opioids may hypersensitize individuals to risk of chronic pain. In addition, our group has shown that opioid-dependent individuals are less likely to employ adaptive coping strategies (118), and report higher chronic stress and traumatic experiences (119) which may further exacerbate hyperalgesic responses. As we have also reviewed, self-regulation of pain relies on neural mechanisms involved in adaptive coping. Thus, chronic opioid use may in fact simultaneously exacerbate the nociceptive aspect of pain while inhibiting individuals’ ability to self-regulate pain. Notably, opioids may be creating a feed-forward cycle that ultimately worsens, rather than alleviates, the experience of chronic pain (shown in Figure 4).

Alternatives to opioid treatment include medications that alter neurotransmitter signaling upstream of, downstream of, or in parallel with endogenous opioid pain-gating (120). For example, serotonin noradrenergic reuptake inhibitors (SNRIs) are often used to treat chronic pain comorbid with anxiety and emotional distress (121–123). Gabapentinoids, whose effect is mediated directly by binding to voltage-gated calcium channels, but perhaps indirectly by altering GABAergic and glutamatergic activity (124–126), are first-line treatment for neuropathic pain (121, 127). Additionally, cannabinoid agonists are rising in popularity for pain treatment, and self-motivated use of medicinal cannabis for pain and stress relief is increasing in the US (128, 129). There is also some evidence that tetrahydrocannabinol can reduce dysfunctional corticomesolimbic connectivity in those with chronic pain (130). However, there is a desperate need for testing of medicinal cannabis products that are non-addictive and for novel non-addictive agents and approaches for pain treatment. As more analgesic agents are introduced, it is crucial that we understand how these substances may interact with the pain circuitry in the brain, and especially pain regulatory pathways of the VmPFC and striatal circuits and their impact on the risk of developing chronic pain. In addition, pain management combined with stress management will be beneficial for the treatment for chronic pain given the substantial overlap between stress and pain pathways. Recent work has shown the effectiveness of mindfulness-based stress reduction, acceptance and commitment therapy (ACT) and cognitive behavioral therapy in chronic pain treatment (131–133). Both psychological strategies and carefully studied pharmacologic solutions may ultimately provide more successful pathways to managing chronic pain without exacerbating the condition or creating new adversities.

## References

[1] Dahlhamer J Lucas J Zelaya C Nahin R Mackey S DeBar L Prevalence of chronic pain and high-impact chronic pain among adults—United States, 2016. MMWR Morb Mortal Wkly Rep. (2018) 67(36):1001–6. 10.15585/mmwr.mm6736a2 30212442 PMC6146950

[2] Treede RD Rief W Barke A Aziz Q Bennett MI Benoliel R A classification of chronic pain for ICD-11. Pain. (2015) 156(6):1003–7. 10.1097/j.pain.0000000000000160 25844555 PMC4450869

[3] Deyo RA Dworkin SF Amtmann D Andersson G Borenstein D Carragee E Focus article report of the NIH task force on research standards for chronic low back pain. Clin J Pain. (2014) 30(8):701–12. 10.1097/AJP.0000000000000120 24988192

[4] Gureje O Von Korff M Kola L Demyttenaere K He Y Posada-Villa J The relation between multiple pains and mental disorders: results from the world mental health surveys. Pain. (2008) 135(1–2):82–91. 10.1016/j.pain.2007.05.005 17570586

[5] Gureje O Simon GE Von Korff M. A cross-national study of the course of persistent pain in primary care. Pain. (2001) 92(1–2):195–200. 10.1016/S0304-3959(00)00483-8 11323140

[6] Frumkin MR Rodebaugh TL. The role of affect in chronic pain: a systematic review of within-person symptom dynamics. J Psychosom Res. (2021) 147:110527. 10.1016/j.jpsychores.2021.110527 34082154 PMC9009535

[7] Abdallah CG Geha P. Chronic pain and chronic stress: two sides of the same coin? Chronic Stress (Thousand Oaks). (2017) 1. 10.1177/2470547017704763 PMC5546756 28795169

[8] Daubresse M Chang HY Yu Y Viswanathan S Shah ND Stafford RS Ambulatory diagnosis and treatment of nonmalignant pain in the United States, 2000–2010. Med Care. (2013) 51(10):870–8. 10.1097/MLR.0b013e3182a95d86 24025657 PMC3845222

[9] Chou R Turner JA Devine EB Hansen RN Sullivan SD Blazina I The effectiveness and risks of long-term opioid therapy for chronic pain: a systematic review for a national institutes of health pathways to prevention workshop. Ann Intern Med. (2015) 162(4):276–86. 10.7326/M14-2559 25581257

[10] Han B Compton WM Blanco C Jones CM. Correlates of prescription opioid use, misuse, use disorders, and motivations for misuse among US adults. J Clin Psychiatry. (2018) 79(5). 10.4088/JCP.17m11973 30153405

[11] Emerson K Boggero I Ostir G Jayawardhana J. Pain as a risk factor for loneliness among older adults. J Aging Health. (2018) 30(9):1450–61. 10.1177/0898264317721348 28728466

[12] Fillingim RB. Sex, gender, and pain. In: Colvin L, Rowbotham DJ, editors. Principles of gender-specific medicine: Gender in the genomic era. 3rd ed. London; San Diego, CA: Elsevier/Academic Press (2017). p. 481–96.

[13] Koons AL Rayl Greenberg M Cannon RD Beauchamp GA. Women and the experience of pain and opioid use disorder: a literature-based commentary. Clin Ther. (2018) 40(2):190–6. 10.1016/j.clinthera.2017.12.016 29329750

[14] Sorge RE Totsch SK. Sex differences in pain. J Neurosci Res. (2017) 95(6):1271–81. 10.1002/jnr.23841 27452349

[15] Rosen S Ham B Mogil JS. Sex differences in neuroimmunity and pain. J Neurosci Res. (2017) 95(1–2):500–8. 10.1002/jnr.23831 27870397

[16] Fillingim RB King CD Ribeiro-Dasilva MC Rahim-Williams B Riley JL. 3rd. Sex, gender, and pain: a review of recent clinical and experimental findings. J Pain. (2009) 10(5):447–85. 10.1016/j.jpain.2008.12.001 19411059 PMC2677686

[17] Nicotra L Tuke J Grace PM Rolan PE Hutchinson MR. Sex differences in mechanical allodynia: how can it be preclinically quantified and analyzed? Front Behav Neurosci. (2014) 8:40. 10.3389/fnbeh.2014.00040 24592221 PMC3923156

[18] Vacca V Marinelli S Pieroni L Urbani A Luvisetto S Pavone F. Higher pain perception and lack of recovery from neuropathic pain in females: a behavioural, immunohistochemical, and proteomic investigation on sex-related differences in mice. Pain. (2014) 155(2):388–402. 10.1016/j.pain.2013.10.027 24231652

[19] Gregory NS Gibson-Corley K Frey-Law L Sluka KA. Fatigue-enhanced hyperalgesia in response to muscle insult: induction and development occur in a sex-dependent manner. Pain. (2013) 154(12):2668–76. 10.1016/j.pain.2013.07.047 23906552 PMC3957416

[20] Kest B Palmese C Hopkins E. A comparison of morphine analgesic tolerance in male and female mice. Brain Res. (2000) 879(1–2):17–22. 10.1016/S0006-8993(00)02685-8 11011001

[21] Fillingim RB Gear RW. Sex differences in opioid analgesia: clinical and experimental findings. Eur J Pain. (2004) 8(5):413–25. 10.1016/j.ejpain.2004.01.007 15324773

[22] Woodhams SG Sagar DR Burston JJ Chapman V. The role of the endocannabinoid system in pain. Handb Exp Pharmacol. (2015) 227:119–43. 10.1007/978-3-662-46450-2_7 25846617

[23] Blanton HL Barnes RC McHann MC Bilbrey JA Wilkerson JL Guindon J. Sex differences and the endocannabinoid system in pain. Pharmacol Biochem Behav. (2021) 202:173107. 10.1016/j.pbb.2021.173107 33444598 PMC8216879

[24] Ferdousi M Finn DP. Stress-induced modulation of pain: role of the endogenous opioid system. Prog Brain Res. (2018) 239:121–77. 10.1016/bs.pbr.2018.07.002 30314566

[25] Zubieta JK Smith YR Bueller JA Xu Y Kilbourn MR Jewett DM mu-opioid receptor-mediated antinociceptive responses differ in men and women. J Neurosci. (2002) 22(12):5100–7. 10.1523/JNEUROSCI.22-12-05100.2002 12077205 PMC6757760

[26] Farquhar CE Breivogel CS Gamage TF Gay EA Thomas BF Craft RM Sex, THC, and hormones: effects on density and sensitivity of CB1 cannabinoid receptors in rats. Drug Alcohol Depend. (2019) 194:20–7. 10.1016/j.drugalcdep.2018.09.018 30391834 PMC6312486

[27] Fogel JS Kelly TH Westgate PM Lile JA. Sex differences in the subjective effects of oral delta(9)-THC in cannabis users. Pharmacol Biochem Behav. (2017) 152:44–51. 10.1016/j.pbb.2016.01.007 26780348 PMC4947027

[28] Wakley AA Wiley JL Craft RM. Sex differences in antinociceptive tolerance to delta-9-tetrahydrocannabinol in the rat. Drug Alcohol Depend. (2014) 143:22–8. 10.1016/j.drugalcdep.2014.07.029 25131716 PMC4161674

[29] Rani Sagar D Burston JJ Woodhams SG Chapman V. Dynamic changes to the endocannabinoid system in models of chronic pain. Philos Trans R Soc Lond B. (2012) 367(1607):3300–11. 10.1098/rstb.2011.0390 23108548 PMC3481532

[30] Morena M Patel S Bains JS Hill MN. Neurobiological interactions between stress and the endocannabinoid system. Neuropsychopharmacology. (2016) 41(1):80–102. 10.1038/npp.2015.166 26068727 PMC4677118

[31] Bluett RJ Baldi R Haymer A Gaulden AD Hartley ND Parrish WP Endocannabinoid signalling modulates susceptibility to traumatic stress exposure. Nat Commun. (2017) 8:14782. 10.1038/ncomms14782 28348378 PMC5379055

[32] Bluett RJ Gamble-George JC Hermanson DJ Hartley ND Marnett LJ Patel S. Central anandamide deficiency predicts stress-induced anxiety: behavioral reversal through endocannabinoid augmentation. Transl Psychiatry. (2014) 4:e408. 10.1038/tp.2014.53 25004388 PMC4119220

[33] Craner JR Lake ES. Adverse childhood experiences and chronic pain rehabilitation treatment outcomes in adults. Clin J Pain. (2021) 37(5):321–9. 10.1097/AJP.0000000000000924 33830091

[34] Axon DR Le D. Predictors of pain severity among community-dwelling older adults with pain in the United States: findings from a cross-sectional, retrospective study using 2017 medical expenditure panel survey. Medicine (Baltimore). (2021) 100(20):e26011. 10.1097/MD.0000000000026011 34011100 PMC8137030

[35] McEwen BS. Physiology and neurobiology of stress and adaptation: central role of the brain. Physiol Rev. (2007) 87(3):873–904. 10.1152/physrev.00041.2006 17615391

[36] Kozakai Y Hori K Aye-Mon A Okuda H Harada SI Hayashi K The role of peripheral corticotropin-releasing factor signaling in a rat model of stress-induced gastric hyperalgesia. Biochem Biophys Res Commun. (2019) 519(4):797–802. 10.1016/j.bbrc.2019.09.040 31558322

[37] Zhang L Chen C Qi J. Activation of HDAC4 and GR signaling contributes to stress-induced hyperalgesia in the medial prefrontal cortex of rats. Brain Res. (2020) 1747:147051. 10.1016/j.brainres.2020.147051 32783961

[38] al'Absi M. Stress and addiction: when a robust stress response indicates resiliency. Psychosom Med. (2018) 80(1):2–16. 10.1097/PSY.0000000000000520 28834923 PMC5741515

[39] Manuel J Rudolph L Beissner F Neubert TA Dusch M Karst M. Traumatic events, posttraumatic stress disorder, and central sensitization in chronic pain patients of a German university outpatient pain clinic. Psychosom Med. (2023) 85(4):351–7. 10.1097/PSY.0000000000001181 36825929 PMC10171308

[40] Ide S Satoyoshi H Minami M Satoh M. Amelioration of the reduced antinociceptive effect of morphine in the unpredictable chronic mild stress model mice by noradrenalin but not serotonin reuptake inhibitors. Mol Pain. (2015) 11:47. 10.1186/s12990-015-0051-0 26260446 PMC4531527

[41] Groenewald CB Murray CB Palermo TM. Adverse childhood experiences and chronic pain among children and adolescents in the United States. Pain Rep. (2020) 5(5):e839. 10.1097/PR9.0000000000000839 32903388 PMC7431222

[42] Achenbach J Rhein M Gombert S Meyer-Bockenkamp F Buhck M Eberhardt M Childhood traumatization is associated with differences in TRPA1 promoter methylation in female patients with multisomatoform disorder with pain as the leading bodily symptom. Clin Epigenetics. (2019) 11(1):126. 10.1186/s13148-019-0731-0 31455424 PMC6712620

[43] First MB Gibbon M Williams JBW. Structured clinical interview for DSM-IV axis I disorders. New York, NY: New York State Psychiatric Institute (1996).

[44] Turner RJ Wheaton B Lloyd DA. The epidemiology of social stress. Am Sociol Rev. (1995) 60:104–25. 10.2307/2096348

[45] Abramson JH. The cornell medical index as an epidemiological tool. Am J Public Health Nations Health. (1966) 56(2):287–98. 10.2105/AJPH.56.2.287 5948222 PMC1256865

[46] Ansell EB Rando K Tuit K Guarnaccia J Sinha R. Cumulative adversity and smaller gray matter volume in medial prefrontal, anterior cingulate, and insula regions. Biol Psychiatry. (2012) 72(1):57–64. 10.1016/j.biopsych.2011.11.022 22218286 PMC3391585

[47] Costa PT Jr McCrae RR. Hypochondriasis, neuroticism, and aging. When are somatic complaints unfounded? Am Psychol. (1985) 40(1):19–28. 10.1037/0003-066X.40.1.19 3977166

[48] Perlmutter M Nyquist L. Relationships between self-reported physical and mental health and intelligence performance across adulthood. J Gerontol. (1990) 45(4):P145–55. 10.1093/geronj/45.4.P145 2365970

[49] Heim C Nemeroff CB. The role of childhood trauma in the neurobiology of mood and anxiety disorders: preclinical and clinical studies. Biol Psychiatry. (2001) 49(12):1023–39. 10.1016/S0006-3223(01)01157-X 11430844

[50] Norman RE Byambaa M De R Butchart A Scott J Vos T. The long-term health consequences of child physical abuse, emotional abuse, and neglect: a systematic review and meta-analysis. PLoS Med. (2012) 9(11):e1001349. 10.1371/journal.pmed.1001349 23209385 PMC3507962

[51] Seo D Rabinowitz AG Douglas RJ Sinha R. Limbic response to stress linking life trauma and hypothalamus-pituitary-adrenal axis function. Psychoneuroendocrinology. (2019) 99:38–46. 10.1016/j.psyneuen.2018.08.023 30172968 PMC6436805

[52] Gerrits MM van Marwijk HW van Oppen P van der Horst H Penninx BW. Longitudinal association between pain, and depression and anxiety over four years. J Psychosom Res. (2015) 78(1):64–70. 10.1016/j.jpsychores.2014.10.011 25466385

[53] Goesling J Clauw DJ Hassett AL. Pain and depression: an integrative review of neurobiological and psychological factors. Curr Psychiatry Rep. (2013) 15(12):421. 10.1007/s11920-013-0421-0 24214740

[54] Ji MJ Yang J Gao ZQ Zhang L Liu C. The role of the kappa opioid system in comorbid pain and psychiatric disorders: function and implications. Front Neurosci. (2021) 15:642493. 10.3389/fnins.2021.642493 33716658 PMC7943636

[55] Scherrer JF Salas J Copeland LA Stock EM Ahmedani BK Sullivan MD Prescription opioid duration, dose, and increased risk of depression in 3 large patient populations. Ann Fam Med. 2016;14(1):54–62. 10.1370/afm.1885 26755784 PMC4709156

[56] Halbert BT Davis RB Wee CC. Disproportionate longer-term opioid use among U.S. adults with mood disorders. Pain. (2016) 157(11):2452–7. 10.1097/j.pain.0000000000000650 27472400 PMC5069117

[57] Sullivan MJ Thorn B Haythornthwaite JA Keefe F Martin M Bradley LA Theoretical perspectives on the relation between catastrophizing and pain. Clin J Pain. (2001) 17(1):52–64. 10.1097/00002508-200103000-00008 11289089

[58] Birch S Stilling M Mechlenburg I Hansen TB. The association between pain catastrophizing, physical function and pain in a cohort of patients undergoing knee arthroplasty. BMC Musculoskelet Disord. (2019) 20(1):421. 10.1186/s12891-019-2787-6 31511076 PMC6739909

[59] Beneciuk JM Bishop MD George SZ. Pain catastrophizing predicts pain intensity during a neurodynamic test for the median nerve in healthy participants. Man Ther. (2010) 15(4):370–5. 10.1016/j.math.2010.02.008 20359935 PMC2893263

[60] Burns LC Ritvo SE Ferguson MK Clarke H Seltzer Z Katz J. Pain catastrophizing as a risk factor for chronic pain after total knee arthroplasty: a systematic review. J Pain Res. (2015) 8:21–32. 10.2147/JPR.S64730 25609995 PMC4294690

[61] Jensen MP Ehde DM Hoffman AJ Patterson DR Czerniecki JM Robinson LR. Cognitions, coping and social environment predict adjustment to phantom limb pain. Pain. (2002) 95(1–2):133–42. 10.1016/S0304-3959(01)00390-6 11790476

[62] Khan RS Ahmed K Blakeway E Skapinakis P Nihoyannopoulos L Macleod K Catastrophizing: a predictive factor for postoperative pain. Am J Surg. (2011) 201(1):122–31. 10.1016/j.amjsurg.2010.02.007 20832052

[63] Unruh AM. Gender variations in clinical pain experience. Pain. (1996) 65(2–3):123–67. 10.1016/0304-3959(95)00214-6 8826503

[64] El-Shormilisy N Strong J Meredith PJ. Associations between gender, coping patterns and functioning for individuals with chronic pain: a systematic review. Pain Res Manag. (2015) 20(1):48–55. 10.1155/2015/490610 24927488 PMC4325891

[65] Darnall BD Sturgeon JA Cook KF Taub CJ Roy A Burns JW Development and validation of a daily pain catastrophizing scale. J Pain. (2017) 18(9):1139–49. 10.1016/j.jpain.2017.05.003 28528981 PMC5581222

[66] Briest J Bethge M. The impact of catastrophizing on the effect of depression on pain and functional ability: a longitudinal mediator analysis. Schmerz. (2017) 31(2):159–66. 10.1007/s00482-016-0172-z 27858219

[67] Higgins C Smith BH Matthews K. Evidence of opioid-induced hyperalgesia in clinical populations after chronic opioid exposure: a systematic review and meta-analysis. Br J Anaesth. (2019) 122(6):e114–26. 10.1016/j.bja.2018.09.019 30915985

[68] Shurman J Koob GF Gutstein HB. Opioids, pain, the brain, and hyperkatifeia: a framework for the rational use of opioids for pain. Pain Med. (2010) 11(7):1092–8. 10.1111/j.1526-4637.2010.00881.x 20545871 PMC2907890

[69] Koob GF. Drug addiction: hyperkatifeia/negative reinforcement as a framework for medications development. Pharmacol Rev. (2021) 73(1):163–201. 10.1124/pharmrev.120.000083 33318153 PMC7770492

[70] Beauchamp GA Nelson LS Perrone J Lyons MS. A theoretical framework and nomenclature to characterize the iatrogenic contribution of therapeutic opioid exposure to opioid induced hyperalgesia, physical dependence, and opioid use disorder. Am J Drug Alcohol Abuse. (2020) 46(6):671–83. 10.1080/00952990.2020.1778713 32897113

[71] Christie NC. The role of social isolation in opioid addiction. Soc Cogn Affect Neurosci. (2021) 16(7):645–56. 10.1093/scan/nsab029 33681992 PMC8259283

[72] Cucinello-Ragland JA Edwards S. Neurobiological aspects of pain in the context of alcohol use disorder. Int Rev Neurobiol. (2021) 157:1–29. 10.1016/bs.irn.2020.09.001 33648668 PMC8356551

[73] LeBlanc DM McGinn MA Itoga CA Edwards S. The affective dimension of pain as a risk factor for drug and alcohol addiction. Alcohol. (2015) 49(8):803–9. 10.1016/j.alcohol.2015.04.005 26008713 PMC4628900

[74] Kesner AJ Lovinger DM. Cannabis use, abuse, and withdrawal: cannabinergic mechanisms, clinical, and preclinical findings. J Neurochem. (2021) 157(5):1674–96. 10.1111/jnc.15369 33891706 PMC9291571

[75] McGowan PO Sasaki A D'Alessio AC Dymov S Labonte B Szyf M Epigenetic regulation of the glucocorticoid receptor in human brain associates with childhood abuse. Nat Neurosci. (2009) 12(3):342–8. 10.1038/nn.2270 19234457 PMC2944040

[76] Burke NN Fan CY Trang T. Microglia in health and pain: impact of noxious early life events. Exp Physiol. (2016) 101(8):1003–21. 10.1113/EP085714 27474262

[77] Wager TD Atlas LY Lindquist MA Roy M Woo CW Kross E. An fMRI-based neurologic signature of physical pain. N Engl J Med. (2013) 368(15):1388–97. 10.1056/NEJMoa1204471 23574118 PMC3691100

[78] Woo CW Roy M Buhle JT Wager TD. Distinct brain systems mediate the effects of nociceptive input and self-regulation on pain. PLoS Biol. (2015) 13(1):e1002036. 10.1371/journal.pbio.1002036 25562688 PMC4285399

[79] Vachon-Presseau E. Effects of stress on the corticolimbic system: implications for chronic pain. Prog Neuropsychopharmacol Biol Psychiatry. (2018) 87(Pt B):216–23. 10.1016/j.pnpbp.2017.10.014 29079140

[80] Woo CW Schmidt L Krishnan A Jepma M Roy M Lindquist MA Quantifying cerebral contributions to pain beyond nociception. Nat Commun. (2017) 8:14211. 10.1038/ncomms14211 28195170 PMC5316889

[81] Atlas LY Lindquist MA Bolger N Wager TD. Brain mediators of the effects of noxious heat on pain. Pain. (2014) 155(8):1632–48. 10.1016/j.pain.2014.05.015 24845572 PMC4104234

[82] McConnell PA Garland EL Zubieta JK Newman-Norlund R Powers S Froeliger B. Impaired frontostriatal functional connectivity among chronic opioid using pain patients is associated with dysregulated affect. Addict Biol. (2020) 25(2):e12743. 10.1111/adb.12743 30945801 PMC6776713

[83] Lee M Manders TR Eberle SE Su C D'Amour J Yang R Activation of corticostriatal circuitry relieves chronic neuropathic pain. J Neurosci. (2015) 35(13):5247–59. 10.1523/JNEUROSCI.3494-14.2015 25834050 PMC4380998

[84] Metz AE Yau HJ Centeno MV Apkarian AV Martina M. Morphological and functional reorganization of rat medial prefrontal cortex in neuropathic pain. Proc Natl Acad Sci U S A. (2009) 106(7):2423–8. 10.1073/pnas.0809897106 19171885 PMC2650172

[85] Apkarian AV. Cortical pathophysiology of chronic pain. Novartis Found Symp. (2004) 261:239–45; discussion 45–61. 15469054

[86] Hashmi JA Baliki MN Huang L Baria AT Torbey S Hermann KM Shape shifting pain: chronification of back pain shifts brain representation from nociceptive to emotional circuits. Brain. (2013) 136(Pt 9):2751–68. 10.1093/brain/awt211 23983029 PMC3754458

[87] Radley JJ Arias CM Sawchenko PE. Regional differentiation of the medial prefrontal cortex in regulating adaptive responses to acute emotional stress. J Neurosci. (2006) 26(50):12967–76. 10.1523/JNEUROSCI.4297-06.2006 17167086 PMC6674963

[88] Milad MR Quirk GJ. Neurons in medial prefrontal cortex signal memory for fear extinction. Nature. (2002) 420(6911):70–4. 10.1038/nature01138 12422216

[89] Kim SC Jo YS Kim IH Kim H Choi JS. Lack of medial prefrontal cortex activation underlies the immediate extinction deficit. J Neurosci. (2010) 30(3):832–7. 10.1523/JNEUROSCI.4145-09.2010 20089891 PMC6633106

[90] Etkin A Wager TD. Functional neuroimaging of anxiety: a meta-analysis of emotional processing in PTSD, social anxiety disorder, and specific phobia. Am J Psychiatry. (2007) 164(10):1476–88. 10.1176/appi.ajp.2007.07030504 17898336 PMC3318959

[91] Baliki MN Petre B Torbey S Herrmann KM Huang L Schnitzer TJ Corticostriatal functional connectivity predicts transition to chronic back pain. Nat Neurosci. (2012) 15(8):1117–9. 10.1038/nn.3153 22751038 PMC3411898

[92] Wanigasekera V Lee MC Rogers R Kong Y Leknes S Andersson J Baseline reward circuitry activity and trait reward responsiveness predict expression of opioid analgesia in healthy subjects. Proc Natl Acad Sci U S A. (2012) 109(43):17705–10. 10.1073/pnas.1120201109 23045652 PMC3491480

[93] Sprenger C Eippert F Finsterbusch J Bingel U Rose M Buchel C. Attention modulates spinal cord responses to pain. Curr Biol. (2012) 22(11):1019–22. 10.1016/j.cub.2012.04.006 22608507

[94] Bhanji JP Delgado MR. Perceived control influences neural responses to setbacks and promotes persistence. Neuron. (2014) 83(6):1369–75. 10.1016/j.neuron.2014.08.012 25199702 PMC4169331

[95] Goldin PR McRae K Ramel W Gross JJ. The neural bases of emotion regulation: reappraisal and suppression of negative emotion. Biol Psychiatry. (2008) 63(6):577–86. 10.1016/j.biopsych.2007.05.031 17888411 PMC2483789

[96] Somerville LH Wagner DD Wig GS Moran JM Whalen PJ Kelley WM. Interactions between transient and sustained neural signals support the generation and regulation of anxious emotion. Cerebral Cortex. (2012) 23(1):49–60. 10.1093/cercor/bhr373 22250290 PMC3513951

[97] Maier SF Watkins LR. Role of the medial prefrontal cortex in coping and resilience. Brain Res. (2010) 1355:52–60. 10.1016/j.brainres.2010.08.039 20727864 PMC2967290

[98] Rushworth MF Noonan MP Boorman ED Walton ME Behrens TE. Frontal cortex and reward-guided learning and decision-making. Neuron. (2011) 70(6):1054–69. 10.1016/j.neuron.2011.05.014 21689594

[99] Miller AH Haroon E Felger JC. Therapeutic implications of brain-immune interactions: treatment in translation. Neuropsychopharmacology. (2017) 42(1):334–59. 10.1038/npp.2016.167 27555382 PMC5143492

[100] Yin L Xu X Chen G Mehta ND Haroon E Miller AH Inflammation and decreased functional connectivity in a widely-distributed network in depression: centralized effects in the ventral medial prefrontal cortex. Brain Behav Immun. (2019) 80:657–66. 10.1016/j.bbi.2019.05.011 31078690 PMC6660411

[101] Seo D Lacadie CM Tuit K Hong KI Constable RT Sinha R. Disrupted ventromedial prefrontal function, alcohol craving, and subsequent relapse risk. JAMA Psychiatry. (2013) 70(7):727–39. 10.1001/jamapsychiatry.2013.762 23636842 PMC3788824

[102] Blaine SK Wemm S Fogelman N Lacadie C Seo D Scheinost D Association of prefrontal-striatal functional pathology with alcohol abstinence days at treatment initiation and heavy drinking after treatment initiation. Am J Psychiatry. (2020) 177(11):1048–59. 10.1176/appi.ajp.2020.19070703 32854534 PMC7606814

[103] Paulus MP Tapert SF Schuckit MA. Neural activation patterns of methamphetamine-dependent subjects during decision making predict relapse. Arch Gen Psychiatry. (2005) 62(7):761–8. 10.1001/archpsyc.62.7.761 15997017

[104] Goldstein RZ Tomasi D Rajaram S Cottone LA Zhang L Maloney T Role of the anterior cingulate and medial orbitofrontal cortex in processing drug cues in cocaine addiction. Neuroscience. (2007) 144(4):1153–9. 10.1016/j.neuroscience.2006.11.024 17197102 PMC1852512

[105] Seo D Tsou KA Ansell EB Potenza MN Sinha R. Cumulative adversity sensitizes neural response to acute stress: association with health symptoms. Neuropsychopharmacology. (2014) 39(3):670–80. 10.1038/npp.2013.250 24051900 PMC3895244

[106] Sinha R Lacadie CM Constable RT Seo D. Dynamic neural activity during stress signals resilient coping. Proc Natl Acad Sci U S A. (2016) 113(31):8837–42. 10.1073/pnas.1600965113 27432990 PMC4978278

[107] Metz S Duesenberg M Hellmann-Regen J Wolf OT Roepke S Otte C Blunted salivary cortisol response to psychosocial stress in women with posttraumatic stress disorder. J Psychiatr Res. (2020) 130:112–9. 10.1016/j.jpsychires.2020.07.014 32805520

[108] al'Absi M Ginty AT Lovallo WR. Neurobiological mechanisms of early life adversity, blunted stress reactivity and risk for addiction. Neuropharmacology. (2021) 188:108519. 10.1016/j.neuropharm.2021.108519 33711348 PMC9195251

[109] Lovallo WR Farag NH Sorocco KH Cohoon AJ Vincent AS. Lifetime adversity leads to blunted stress axis reactivity: studies from the Oklahoma family health patterns project. Biol Psychiatry. (2012) 71(4):344–9. 10.1016/j.biopsych.2011.10.018 22112928 PMC3264696

[110] Zhong X Ming Q Dong D Sun X Cheng C Xiong G Childhood maltreatment experience influences neural response to psychosocial stress in adults: an fMRI study. Front Psychol. (2019) 10:2961. 10.3389/fpsyg.2019.02961 31993010 PMC6971063

[111] Seminowicz DA Davis KD. Cortical responses to pain in healthy individuals depends on pain catastrophizing. Pain. (2006) 120(3):297–306. 10.1016/j.pain.2005.11.008 16427738

[112] Kornstein SG Schatzberg AF Thase ME Yonkers KA McCullough JP Keitner GI Gender differences in chronic major and double depression. J Affect Disord. (2000) 60(1):1–11. 10.1016/S0165-0327(99)00158-5 10940442

[113] Kessler RC McGonagle KA Zhao S Nelson CB Hughes M Eshleman S Lifetime and 12-month prevalence of DSM-III-R psychiatric disorders in the United States. Results from the national comorbidity survey. Arch Gen Psychiatry. (1994) 51(1):8–19. 10.1001/archpsyc.1994.03950010008002 8279933

[114] Goldfarb EV Seo D Sinha R. Sex differences in neural stress responses and correlation with subjective stress and stress regulation. Neurobiol Stress. (2019) 11:100177. 10.1016/j.ynstr.2019.100177 31304198 PMC6603439

[115] Kapur BM Lala PK Shaw JL. Pharmacogenetics of chronic pain management. Clin Biochem. (2014) 47(13–14):1169–87. 10.1016/j.clinbiochem.2014.05.065 24912048

[116] Stacey DTaBR. Pharmacologic management of chronic non-cancer pain in adults (2021). Available at: https://www.uptodate.com/contents/pharmacologic-management-of-chronic-non-cancer-pain-in-adults (August 2021).

[117] Beal BR Wallace MS. An overview of pharmacologic management of chronic pain. Med Clin North Am. (2016) 100(1):65–79. 10.1016/j.mcna.2015.08.006 26614720

[118] Hyman SM Hong KI Chaplin TM Dabre Z Comegys AD Kimmerling A A stress-coping profile of opioid dependent individuals entering naltrexone treatment: a comparison with healthy controls. Psychol Addict Behav. (2009) 23(4):613–9. 10.1037/a0017324 20025367 PMC2802459

[119] Barry DT Beitel M Cutter CJ Garnet B Joshi D Rosenblum A Exploring relations among traumatic, posttraumatic, and physical pain experiences in methadone-maintained patients. J Pain. (2011) 12(1):22–8. 10.1016/j.jpain.2010.04.006 20646965 PMC2962776

[120] Tracey I Mantyh PW. The cerebral signature for pain perception and its modulation. Neuron. (2007) 55(3):377–91. 10.1016/j.neuron.2007.07.012 17678852

[121] Finnerup NB Attal N Haroutounian S McNicol E Baron R Dworkin RH Pharmacotherapy for neuropathic pain in adults: a systematic review and meta-analysis. Lancet Neurol. (2015) 14(2):162–73. 10.1016/S1474-4422(14)70251-0 25575710 PMC4493167

[122] Arnold LM. Duloxetine and other antidepressants in the treatment of patients with fibromyalgia. Pain Med. (2007) 8(Suppl 2):S63–74. 10.1111/j.1526-4637.2006.00178.x 17714117

[123] Pergolizzi JV Jr Raffa RB Taylor R Jr Rodriguez G Nalamachu S Langley P. A review of duloxetine 60 mg once-daily dosing for the management of diabetic peripheral neuropathic pain, fibromyalgia, and chronic musculoskeletal pain due to chronic osteoarthritis pain and low back pain. Pain Pract. (2013) 13(3):239–52. 10.1111/j.1533-2500.2012.00578.x 22716295

[124] Yoshizumi M Parker RA Eisenach JC Hayashida K. Gabapentin inhibits gamma-amino butyric acid release in the locus coeruleus but not in the spinal dorsal horn after peripheral nerve injury in rats. Anesthesiology. (2012) 116(6):1347–53. 10.1097/ALN.0b013e318254e6fd 22487864 PMC3360795

[125] Kremer M Salvat E Muller A Yalcin I Barrot M. Antidepressants and gabapentinoids in neuropathic pain: mechanistic insights. Neuroscience. (2016) 338:183–206. 10.1016/j.neuroscience.2016.06.057 27401055

[126] Taylor CP. Mechanisms of analgesia by gabapentin and pregabalin–calcium channel alpha2-delta [Cavalpha2-delta] ligands. Pain. (2009) 142(1–2):13–6. 10.1016/j.pain.2008.11.019 19128880

[127] Wiffen PJ DS Moore RA Aldington D Cole P Rice AS Lunn MP Ntiepileptic drugs for neuropathic pain and fibromyalgia—an overview of cochrane reviews. Cochrane Database Syst Rev. (2013) 2013(11):CD010567. 24217986 10.1002/14651858.CD010567.pub2 PMC6469538

[128] Azcarate PM Zhang AJ Keyhani S Steigerwald S Ishida JH Cohen BE. Medical reasons for marijuana use, forms of use, and patient perception of physician attitudes among the US population. J Gen Intern Med. (2020) 35(7):1979–86. 10.1007/s11606-020-05800-7 32291715 PMC7352011

[129] Karst M Wippermann S Ahrens J. Role of cannabinoids in the treatment of pain and (painful) spasticity. Drugs. (2010) 70(18):2409–38. 10.2165/11585260-000000000-00000 21142261

[130] Weizman L Dayan L Brill S Nahman-Averbuch H Hendler T Jacob G Cannabis analgesia in chronic neuropathic pain is associated with altered brain connectivity. Neurology. (2018) 91(14):e1285–94. 10.1212/WNL.0000000000006293 30185448 PMC6177269

[131] Cherkin DC Sherman KJ Balderson BH Cook AJ Anderson ML Hawkes RJ Effect of mindfulness-based stress reduction vs cognitive behavioral therapy or usual care on back pain and functional limitations in adults with chronic low back pain: a randomized clinical trial. JAMA. (2016) 315(12):1240–9. 10.1001/jama.2016.2323 27002445 PMC4914381

[132] McBeth J Prescott G Scotland G Lovell K Keeley P Hannaford P Cognitive behavior therapy, exercise, or both for treating chronic widespread pain. Arch Intern Med. (2012) 172(1):48–57. 10.1001/archinternmed.2011.555 22082706

[133] McCracken LM Yu L Vowles KE. New generation psychological treatments in chronic pain. Br Med J. (2022) 376:e057212. 10.1136/bmj-2021-057212 35228207

